# Cancer survival and its social determinants among children with a migrant background: systematic review protocol

**DOI:** 10.1136/bmjopen-2026-116611

**Published:** 2026-05-04

**Authors:** Vian Rajabzadeh, Abla Sami, Arja H Harila, David A Richards, Joanne Woodford, Louise von Essen

**Affiliations:** 1 CIRCLE – Complex Intervention Research in Health and Care, Department of Women’s and Children’s Health, Uppsala University, Uppsala, Sweden; 2 Wolfson Institute of Population Health, Queen Mary University of London, London, UK; 3 Department of Women’s and Children’s Health, Uppsala University, Uppsala, Sweden; 4 Department of Health and Caring Sciences, Western Norway University of Applied Sciences, Bergen, Norway

**Keywords:** Paediatric oncology, Survival, Health Equity

## Abstract

**Abstract:**

**Introduction:**

Childhood cancer survival rates are not equal for all, with disparities existing both between and within low- and middle-income countries and high-income countries, particularly among ethnic minorities and children with migrant backgrounds. Factors such as cultural misunderstandings, language barriers and limited support networks can lead to delays in diagnosis and treatment challenges, which can result in poor health outcomes. Social determinants of health (SDoH), such as housing insecurity and poverty, may worsen these disparities. This protocol outlines a systematic review to examine childhood cancer survival in children with migrant backgrounds compared with non-migrants and to explore the SDoH associated with these survival outcomes.

**Methods and analysis:**

We will search MEDLINE (PubMed), Scopus, Web of Science, and Embase for relevant studies, with secondary searches of grey literature. Two reviewers will screen for observational studies, including longitudinal cohort, case–control, cross-sectional and registry-based studies, that report childhood cancer survival outcomes (eg, survival rates, HRs) for both migrant and non-migrant populations. A narrative synthesis will explore SDoH and their association with survival outcomes. If data allow, we will perform random-effects meta-analyses to estimate pooled survival outcomes. Subgroup analyses will examine factors such as geographic region, migration status and type of cancer.

**Discussion:**

Understanding factors contributing to childhood cancer survival disparities in migrant populations is critical for informing the development of targeted strategies to address them, ensuring all children, regardless of their migration status, have an equitable opportunity for effective care and improved outcomes.

**Ethics and dissemination:**

Ethical approval is not required for this study as it is based on previously published data and does not involve primary data collection. We will publish the results in peer-reviewed journals and present at academic conferences.

**PROSPERO registration number:**

CRD42024547239.

**STRENGTHS AND LIMITATIONS OF THIS STUDY**

- The review protocol adheres to rigorous methodological standards, including Joanna Briggs Institute and Preferred Reporting Items for Systematic Reviews and Meta-Analyses guidelines.
- The search strategy has been peer-reviewed to ensure rigour and completeness.
- The comprehensive search strategy includes electronic databases, grey literature and manual searches to find a wide range of relevant studies and reduce selection bias.
- If feasible, we will conduct a meta-analysis and subgroup analyses to generate more reliable and generalisable findings by pooling data.
- Primary limitations include the exclusion of purely qualitative studies, which may limit patient perspectives, and a potential language bias from including only English-language publications.

## Introduction

 Despite the advancement in treatment and post-treatment care, childhood cancer remains the leading global and public health challenge.^1 2^ Every year, about 400 000 children globally are diagnosed with cancer, primarily leukaemia and brain tumour.^3 4^ Survival outcomes for children with cancer vary between countries, with high-income countries (HICs) reporting survival rates of 80%, while in low- and middle-income countries (LMICs) survival rates can be as low as 20%.^5 6^ Factors such as delayed diagnosis, inadequate healthcare infrastructure, limited access to treatment and malnutrition contribute to these disparities.^7^,^9^ Financial hardship, high treatment costs, insufficient knowledge of symptoms among parents and caregivers and systemic biases—such as healthcare policies that prioritise certain groups over others—can further compound these challenges.^79^,^12^ These disparities are observed across many LMIC settings. For example, in sub-Saharan Africa survival rates for Wilms tumour are 11% in Sudan and 46% in Malawi, while they range from 70% to 97% in HICs,^13^ primarily due to low treatment adherence rates.^14 15^ Similarly, evidence from Asia highlights substantial variation in survival across countries, with survival reported to be as low as 10% in the Philippines and 5% in Bangladesh.^16^

While disparities in childhood cancer survival are most pronounced in LMICs, inequalities persist even within HICs.^17^ These inequalities are often driven by social determinants of health (SDoH), for example, factors such as inadequate housing, limited transportation access, poor living conditions and poverty.^18^,^21^ A recent scoping review by Majercak *et al* identified several key SDoH that impact health equity in oncology community settings.^22^ The review highlighted that factors such as higher household earnings, non-discrimination, social inclusion and social protection are linked to better health outcomes.^22^ Conversely, factors like structural conflict, for example, societal and institutional clashes such as war, political instability or other forms of sustained violence, are associated with worse outcomes, such as lower survival rates and quality of life.^22^ Studies in countries with universal healthcare, including Denmark, Sweden, Switzerland and the UK, have identified disparities in childhood cancer survival based on socioeconomic factors.^23^,^27^ Both adults and children from ethnic minority groups have been found to experience low survival rates, especially when socioeconomic status is considered.^1923 25 26 28^,^31^ For example, a UK study found that children with lymphoma from a South Asian background had a 15% reduced chance of survival at 5 years compared with non-South Asian children, and children living in the most deprived areas had a 1.11-fold increased risk of death.^25^

Research also suggests disparities in childhood cancer survival may be rooted in a complex interplay of factors, including cultural beliefs and influences—such as a mistrust of Western medicine or a reliance on traditional healers, financial strain, health literacy, language barriers, limited access to culturally appropriate healthcare, socioeconomic inequalities and stigma.^2431^,^33^ A recent qualitative study on childhood cancer stigma with caregivers, patients and clinicians across three countries (Guatemala, Jordan and Zimbabwe) found that participants described a shared stigma experience that transcended age, culture, diagnosis and geography.^33^ Despite ongoing research into the relationship between childhood cancer and SDoH, our understanding of the interaction between these factors remains limited, and underlying mechanisms remain unclear.^34^

While ethnicity and SDoH are recognised as being associated with childhood cancer survival, the experiences of migrant children, a growing population worldwide,^35^ may present unique complexities. Migration itself can be viewed as both a consequence of SDoH and an SDoH in its own right.^36^ SDoH, such as cultural competence and the lack of diversity within healthcare workforces, have been found to increase health inequalities among migrant populations in the USA.^24^ A recent scoping review identified persistent disparities in healthcare utilisation among migrants with established legal status in Europe, compared with non-migrants.^37^ Results suggested these disparities were driven by a variety of factors, including discrimination, language difficulties and legal barriers. Additionally, an inefficient pattern of healthcare use was identified—characterised by the underuse of primary care and overuse of emergency services—as further contributing to these inequalities.^35^ The 2022 WHO report on the health of refugees and migrants highlighted significant gaps in cancer knowledge among refugee and migrant children, and limited data available on their health outcomes, including survival.^38^ Poverty is a factor that disproportionately affects migrant children, especially those from refugee backgrounds,^38 39^ and has been found to impact survival rates in childhood cancer.^2440^,^42^ Lower childhood cancer survival rates have also been found among children from low-income and migrant families when compared with those with a non-migrant background across several cancer types in Finland^43^ and Denmark and Sweden.^27^ However, a study from Jordan reported survival outcomes among displaced children with cancer comparable to those of the host population, although findings were based on a single setting and should be interpreted with caution.^44^ Children with cancer depend on their informal caregivers for emotional support, practical assistance and treatment plan adherence. However, caregivers with migrant backgrounds may face cultural misunderstandings, language barriers and limited support networks, which can impact their ability to navigate the healthcare system.^29 42 43 45^ These barriers, compounded with the financial difficulties and the demands of caregiving, can lead to delays in diagnosis, treatment adherence challenges and ultimately, poorer health outcomes for children with cancer from migrant backgrounds.^46^

While research has explored the relationship between SDoH and childhood cancer survival in the general population,^27^ less attention has been paid to examining differences in survival between children with a migrant and non-migrant background. In addition, there is a lack of research exploring SDoH (eg, socioeconomic, sociocultural and healthcare system factors) potentially associated with childhood cancer survival.

### Research aims

- To examine childhood cancer survival outcomes among children (0–19 years at diagnosis) with a migrant background compared with children with a non-migrant background.
- To explore SDoH potentially associated with childhood cancer survival.

## Methods

This protocol adheres to the Preferred Reporting Items for Systematic Review and Meta-Analysis Protocols (PRISMA-P) 2015 guidelines^47^ (online supplemental appendix 1) and was developed using the Joanna Briggs Institute (JBI) methodology.^48^ For reporting the review’s results, we will follow the PRISMA 2020 guidelines. We have registered the protocol on PROSPERO (CRD42024547239), and any subsequent modifications will be documented there and included in the final review. The review is planned to be conducted between August 2024 and January 2027.

### Eligibility criteria

#### Population

We will include studies from anywhere globally that examine childhood cancer survival in participants aged 0–19 years at diagnosis. We define children with a migrant background as individuals who have migrated to a new host country or were born in the host country to at least one parent who had migrated there. Eligible studies will report data on both children with a migrant background and children with a non-migrant background, allowing for a comparison of survival outcomes. Studies that only report data on children with a migrant background, without a comparison, will also be eligible for inclusion and will contribute to the narrative synthesis to help identify SDoH potentially associated with survival outcomes. We will exclude studies that include only adult populations or those unable to provide separate child-specific data.

#### Outcomes

We will include studies reporting survival outcome data for children with cancer, including survival rates (1, 2, 3, 5 and 10-year survival rates), HRs and other survival metrics such as Kaplan-Meier (KM) curves or Cox proportional hazards models.

#### Study design

We will include observational studies examining childhood cancer survival. Eligible study designs include case–control, cross-sectional, longitudinal cohort, mixed methods studies and registry-based studies. We will exclude case reports, case series, editorials, opinions and non-peer-reviewed articles. We will only include studies reported in English-language journals.

#### Publication date

To ensure the review reflects the latest advancements in childhood cancer treatment, our review will focus on studies published since 2000.^49^

### Information sources

We will search four primary electronic databases: MEDLINE (PubMed), Scopus, Web of Science/Web of Knowledge and Embase/Elsevier Science. To enhance the comprehensiveness of the search,^50^ we will search additional sources, including Google Scholar, OpenGrey, the WHO Library Database (WHOLIS), government surveillance data and reports, Centers for Disease Control and Prevention (CDC) data, national health survey data and relevant disease association resources (eg, International Agency for Research on Cancer). We will undertake manual searches through forward and backward citation tracking to identify studies that the electronic database searches may have missed.

### Search strategy

We developed the search strategy in collaboration with library services at Uppsala University, using terms related to paediatrics, cancer, migrants, SDoH and survival outcomes. The strategy combined free-text and Medical Subject Headings (MeSH) terms to maximise search sensitivity and specificity. It was subsequently peer-reviewed by external reviewers (one expert in paediatric oncology and one expert in evidence synthesis and global health), adhering to Peer Review of Electronic Search Strategies (PRESS) guidelines in online supplemental appendix 2.^51^ Due to database-specific indexing variations, we adapted the search strategy for each database. Our initial search included studies with English titles or abstracts. We have provided the search strategy for all databases in online supplemental appendix 3. Updated searches will be conducted within 3 months before submitting the results manuscript.

### Study selection

We will collate all retrieved studies and upload them into Covidence,^52^ where duplicates will be removed. To mitigate duplicate inclusions from registry-based studies, we will apply search filters, use deduplication techniques and contact study authors for clarification as needed.^53^ Two reviewers will independently screen titles and abstracts to determine eligibility, followed by a full-text screening to identify studies for inclusion. We will exclude studies without accessible English full-text versions at this stage. If essential data or information needed to determine eligibility is missing, we will contact the study authors via email for additional information. We will resolve any discrepancies through discussions and/or by involving a third reviewer. Using a PRISMA flowchart, we will report the number of studies screened and excluded. Additionally, to provide greater clarity, we will include a more detailed table of reasons for excluding studies in the online supplemental appendix.

### Data extraction

One reviewer will extract data independently from included studies using a standardised Microsoft Excel data extraction form (online supplemental appendix 4), cross-checked by a second reviewer for accuracy. We will extract the following data:

1. Study identification features: study ID, study title, authors’ names, year of publication, citation, DOI and URL link, publication type or data source (eg, journal or report), and abstract (if available).
2. Study characteristics: aim of the study, setting (eg, geographical region), study design (eg, cohort study, cross-sectional, registry-based studies), study duration or follow-up, population age, sample size, sampling method, method of data analysis, recruitment method, recruitment setting, and type of consent, reported survival rate.
3. Population characteristics will be extracted using the PRO EDI tool ^54^, a standardised participant characteristics table designed to evaluate the reporting and analysis of equity, diversity, and inclusion within randomised trials. This includes detailed information on sex (male-to-female ratio or percentage), gender, age at diagnosis (mean/median age), country of population, type of cancer, the distribution of cancer, type of migration, race/ethnicity and country of origin.
4. Results summary: survival outcomes, including survival rates (1, 2, 3, 5 and 10-year survival rates), HRs, 95% CIs and other survival metrics, such as KM curves.
5. SDoH: we will extract data on SDoH, using the CDC’s SDoH framework,^55^ which categorises SDoH into five domains: Economic Stability, Education Access and Quality, Healthcare Access and Quality, Neighbourhood and Built Environment and Social and Community Context.

### Quality assessment

We will quality assess included studies using the Newcastle Ottawa Scale (NOS).^56^ Two reviewers will independently assess the quality of each study using the NOS, and any discrepancies will be resolved through discussion or consulting a third reviewer. The NOS evaluates studies based on three domains: (1) selection of participants, (2) comparability of study groups, and (3) outcome/exposure. NOS scores range from 0 to 9, with higher scores indicating higher quality. Studies with scores of 7 or more will be considered high quality, those with scores of 5–6 will be considered of moderate quality and those with scores of 4 or fewer will be considered of low quality. To accurately assess the relevance and quality of each study in relation to migrant health, we will pose two additional questions beyond the standard quality assessment criteria. This approach, which has precedent in similar research,^57^ allows us to assess how well each study captures the specific factors that influence childhood cancer survival in migrant populations. The questions are: (1) Was the primary aim of the study to examine childhood cancer survival among migrants? and (2) Did the study examine the influence of migration on childhood cancer survival?

### Data analysis and synthesis

#### Narrative synthesis

Our narrative synthesis aims to review and synthesise all reported survival outcomes. We will critically appraise each included study using the NOS scores. Studies with higher NOS scores will be given more weight in the narrative synthesis, while those with lower NOS scores will be given less weight. This approach will consider factors such as study design, sample size, participant selection methods, data collection procedures, statistical analyses, and potential for bias. If data allow, we will synthesise the potential association between SDoH and childhood cancer survival. We will develop a coding scheme based on the five domains of the CDC SDoH framework.^55^ We will present potential SDoH-survival associations and relevant data from the studies in tables or diagrams to visually summarise key findings within each domain.

#### Meta-analysis

Our decision to conduct a meta-analysis will be based on several key factors. We will assess if there is a sufficient number of studies (minimum of five) to conduct a meta-analysis to account for both within-study error and between-study variance (heterogeneity). With very few studies (eg, 2–4), the estimation of between-study variance is unstable and the meta-analysis may not offer more statistical power than the individual studies themselves.^58^ We will examine the clinical and methodological similarity (ie, acceptable homogeneity) of the included studies’ populations and outcomes. Data availability and quality, as evaluated by our critical appraisal scores, will also inform our decision to conduct a meta-analysis. If it is not possible to conduct a meta-analysis, we will proceed with a narrative synthesis only, and this decision will be fully documented.

If a meta-analysis is deemed feasible, we will compare survival outcomes between migrant and non-migrant children. Ideally, we will pool HRs, as they are the most appropriate effect measure for time-to-event outcome, accounting not only for the number and the timing of the event (eg, death), but also the time until the last follow-up for each participant who did not experience the event during the study period (ie, censored participants).^59 60^

In cases where HRs are not reported, we will estimate them, where possible using updated methods for incorporating summary time-to-event data as described by Tierney *et al*,^60^ for example, HRs and their variance will be derived from reported summary statistics such as log rank test results, event counts or numbers at risk. If KM curves are available, we will estimate the HR using one of the following scenarios. First, if the paper provides a KM curve together with a table of additional information (eg, numbers at risk, total events or median survival times), we will digitise the KM curve and combine these data to improve the accuracy of HR estimation, using established methods to reconstruct summary data.^61^ A survival model will be fitted (such as the Cox proportional hazards model) with the HR calculated using RStudio.^62^ Second, if only the visual KM curve is available, with no numbers at risk or event counts, we will digitise the curve to extract survival probabilities at multiple time points and reconstruct approximate individual patient data using established algorithms.^61^ A Cox proportional hazards model will then be fitted to estimate the HR. Because this approach relies on stronger assumptions (eg, censoring patterns), the estimates will be flagged as approximate and explored in sensitivity analyses. By obtaining HRs from the KM curves, we can ensure a consistent approach to comparing the survival between migrant and non-migrant children across different studies.

If available studies primarily report survival rates instead of HRs, we will explore the feasibility of conducting a meta-analysis on these alternative survival metrics. However, we acknowledge the potential limitations associated with these metrics, such as the limited ability to account for time-varying effects and the potential impact of censoring on the results.

To assess heterogeneity among included studies, we will employ the Cochran test (with a significance level of p<0.1) and calculate the I^2^ statistic. I^2^ values of 25% or less, 50% and 75% or more represent low, moderate and high heterogeneity, respectively.^63^ If heterogeneity is detected among the included studies, we will use a random-effects model with the inverse-variance method.^63^ In the absence of heterogeneity, we will adopt a fixed-effects model. We will examine sources of heterogeneity using mixed-effects regression with unrestricted maximum likelihood estimation.^63^ We will use the prediction interval (T^2^) to estimate between-study variance in true effects. We will conduct a sensitivity analysis to assess the robustness of pooled estimates by evaluating the impact of individual studies on the overall results. Factors considered will include sample size, study design and methodological quality, as assessed by the NOS scoring (see Subgroup analysis below). We will use the trim and fill method to explore the potential influence of publication bias^64^ and use funnel plots to assess the potential for publication bias with the Egger’s regression test, reporting the p value for funnel symmetry.^65^ We will use Comprehensive Meta-Analysis software^66^ to conduct the meta-analysis.

#### Subgroup analysis

If a sufficient number of studies is available and significant heterogeneity is observed, we will perform subgroup analyses to explore factors that may contribute to these differences. This will provide a more nuanced understanding of survival outcomes among children with a migrant background across the included studies. Subgroup analysis will be performed (if data allow) on the following factors: (1) geographical region (following the United Nations region classification,^67^ which includes Africa, Asia-Pacific, Eastern Europe, Latin America and the Caribbean, Northern America and Western Europe and Others); (2) host country income (categorised according to the World Bank classification^68^ into low-income economies, lower-middle-income economies, upper-middle-income economies and high-income economies); (3) migrant status (classified based on the type of migration, which includes refugees and asylum seekers, international students, migrant workers, family migration, irregular migrants, stateless persons and internally displaced persons)^69^,^71^; (4) type of cancer (eg, leukaemia, lymphomas); (5) study design (eg, case–control studies, cross-sectional studies and longitudinal cohort studies); and (6) sample size (eg, <100 and ≥100); and (7) NOS score (eg, 0–9).

### Patient and public involvement

Patients or members of the public were not involved in designing this systematic review due to limited resources. However, we recognise the importance of involving patients and members of under-represented communities in research.^72^ We acknowledge some of the authors are themselves migrants and have lived experience of being a family member of a child with cancer, which provided valuable perspectives into the design of this systematic review, but recognise this does not replace formal patient and public involvement.

## Discussion

Despite research on childhood cancer survival and disparities in HICs, less attention has been paid to examining differences in survival between children with migrant and non-migrant backgrounds and the potential influence of SDoH.^17^ To our knowledge, no systematic review has compared childhood cancer survival between migrant and non-migrant populations or explored SDoH potentially associated with survival. This gap highlights the urgent need for research to identify specific factors driving these disparities and to inform targeted interventions that can improve survival outcomes for migrant children with cancer.

Our systematic review has several notable strengths. We will adhere to rigorous methodological standards outlined by JBI guidelines^48^ and employ a comprehensive search strategy to maximise inclusivity. The search will cover electronic databases, grey literature and manual searches, including forward and backward citation tracking. Our search strategy underwent a peer review by external experts in accordance with the PRESS guidelines, ensuring its rigour and completeness.^51^ It also aligns with PRISMA reporting guidelines to ensure transparency and replicability. To minimise the risk of duplicate studies, particularly those using registry-based data, we will employ rigorous search filters, de-duplication techniques and contact study authors for clarification as needed.

By incorporating a thorough list of SDoH and related terms in the electronic database searches, we aim to identify a broader range of studies that might indirectly address migrant populations, thereby increasing the potential to uncover valuable data. To explore the potential associations between SDoH and childhood cancer survival, we will perform a narrative synthesis. The NOS will be used to assess the methodological quality of included studies. We will assess heterogeneity and NOS scores to determine the feasibility of conducting a meta-analysis. If data allow, we will perform a meta-analysis and subgroup analyses to generate more reliable and generalisable findings by pooling data from various sources. Funnel plots will be used to evaluate publication bias.

This systematic review has several limitations. By excluding qualitative studies, we will not be able to explore the perspectives of patients and caregivers on how SDoH may influence childhood cancer survival. Limiting the review to English-language publications may introduce a language bias, potentially excluding relevant research conducted in languages other than English.

Due to the limited representation of migrant children in childhood cancer research^17 43 73 74^ and existing healthcare inequalities that contribute to survival disparities, urgent action is needed.^73^ By comparing survival outcomes between migrant and non-migrant children, we seek to identify potential survival disparities. Findings may inform evidence-based strategies such as developing culturally competent clinical guidelines and guiding public health policies to improve outcomes for this population.^17^ Our work contributes to global health initiatives, aligning with key Sustainable Development Goals (SDGs), including SDG 3 (Good Health and Well-being) and SDG 10 (Reduced Inequalities), by addressing health disparities in marginalised populations.

## Ethics and dissemination

While a systematic review does not require ethical approval, we will incorporate ethical considerations throughout our review process, guided by established principles.^75^ We will examine included studies for ethical approval and informed consent. We will proactively identify potential ethical concerns, such as inadequate data protection or a risk of stigmatisation. When such issues are found, we will address them by critically discussing these limitations in our findings. If a concern presents a significant risk of bias (eg, selection bias, incomplete data or HR estimated under a high level of uncertainty), we will perform a sensitivity analysis by re-running the meta-analysis without the study to assess its impact on the results.

We are committed to minimising bias in our analysis and interpretation. This will be achieved by strictly following a preregistered protocol that details our search strategy, inclusion criteria and statistical methods. We will also use established tools to assess the risk of bias in each study and account for this in our analysis. We will adhere to transparent reporting guidelines, such as the PRISMA statement,^76^ and will make our data and materials publicly available to promote reproducibility.

The results of this study will be disseminated through publications in peer-reviewed journals and presentation at relevant national and international academic conferences.

## Supplementary material

10.1136/bmjopen-2026-116611 online supplemental file 1
