# Supplementary material for: Cancer survival and its social determinants among children with a migrant background: systematic review protocol
Source: BMJ Open. 2026 May 4;16(5):e116611. doi: 10.1136/bmjopen-2026-116611 (PMC13141170; doi:10.1136/bmjopen-2026-116611)
Supplement: online supplemental file 1 [file bmjopen-16-5-s001.pdf]

Supplementary Appendix 1: PRISMA-P

PRISMA-P (Preferred Reporting Items for Systematic review and Meta-Analysis Protocols) 2015 checklist: Recommended items to address in a systematic review protocol*

Section and topic	Item No	Checklist item	Location in text (page number)
**ADMINISTRATIVE INFORMATION**			
Title:			
Identification	1a	Identify the report as a protocol of a systematic review	Title page
Update	1b	If the protocol is for an update of a previous systematic review, identify as such	NA
Registration	2	If registered, provide the name of the registry (such as PROSPERO) and registration number	2& 5
Authors:			
Contact	3a	Provide name, institutional affiliation, e-mail address of all protocol authors; provide physical mailing address of corresponding author	Title page
Contributions	3b	Describe contributions of protocol authors and identify the guarantor of the review	14-15
Amendments	4	If the protocol represents an amendment of a previously completed or published protocol, identify as such and list changes; otherwise, state plan for documenting important protocol amendments	NA
Support:			
Sources	5a	Indicate sources of financial or other support for the review	15
Sponsor	5b	Provide name for the review funder and/or sponsor	15
Role of sponsor or funder	5c	Describe roles of funder(s), sponsor(s), and/or institution(s), if any, in developing the protocol	15
**INTRODUCTION**			
Rationale	6	Describe the rationale for the review in the context of what is already known	1-4
Objectives	7	Provide an explicit statement of the question(s) the review will address with reference to participants, interventions, comparators, and outcomes (PICO)	5
**METHODS**			
Eligibility criteria	8	Specify the study characteristics (such as PICO, study design, setting, time frame) and report characteristics (such as years considered, language, publication status) to be used as criteria for eligibility for the review	5-6

Information sources	9	Describe all intended information sources (such as electronic databases, contact with study authors, trial registers or other grey literature sources) with planned dates of coverage	6-7
Search strategy	10	Present draft of search strategy to be used for at least one electronic database, including planned limits, such that it could be repeated	6-7
Study records:			
Data management	11a	Describe the mechanism(s) that will be used to manage records and data throughout the review	7-8
Selection process	11b	State the process that will be used for selecting studies (such as two independent reviewers) through each phase of the review (that is, screening, eligibility and inclusion in meta-analysis)	7
Data collection process	11c	Describe planned method of extracting data from reports (such as piloting forms, done independently, in duplicate), any processes for obtaining and confirming data from investigators	7-8
Data items	12	List and define all variables for which data will be sought (such as PICO items, funding sources), any pre-planned data assumptions and simplifications	7-8
Outcomes and prioritization	13	List and define all outcomes for which data will be sought, including prioritization of main and additional outcomes, with rationale	6-8
Risk of bias in individual studies	14	Describe anticipated methods for assessing risk of bias of individual studies, including whether this will be done at the outcome or study level, or both; state how this information will be used in data synthesis	8-9
Data synthesis	15a	Describe criteria under which study data will be quantitatively synthesised	9-12
	15b	If data are appropriate for quantitative synthesis, describe planned summary measures, methods of handling data and methods of combining data from studies, including any planned exploration of consistency (such as I^2, Kendall's τ)	9-12
	15c	Describe any proposed additional analyses (such as sensitivity or subgroup analyses, meta-regression)	9-12
	15d	If quantitative synthesis is not appropriate, describe the type of summary planned	9
Meta-bias(es)	16	Specify any planned assessment of meta-bias(es) (such as publication bias across studies, selective reporting within studies)	11
Confidence in cumulative evidence	17	Describe how the strength of the body of evidence will be assessed (such as GRADE)	9

Supplementary Appendix 2: PRESS Peer Review

PRESS Guideline — Search Submission & Peer Review Assessment

SEARCH SUBMISSION: *THIS SECTION TO BE FILLED IN BY THE SEARCHER*

Searcher: Vian Rajabzadeh **Email:** vian.rajabzadeh.uu.se
Reviewer: 1
Date submitted: Date requested by: 5th August 2024

Systematic Review Title:

Childhood Cancer Survival among Children with a Migrant Background: A Systematic Review Protocol

This search strategy is …

x	My PRIMARY (core) database strategy — First time submitting a strategy for search question and database
	My PRIMARY (core) strategy — Follow-up review NOT the first time submitting a strategy for search question and database. If this is a response to peer review, itemize the changes made to the review suggestions
	SECONDARY search strategy— First time submitting a strategy for search question and database
	SECONDARY search strategy — NOT the first time submitting a strategy for search question and database. If this is a response to peer review, itemize the changes made to the review suggestions

Database

(i.e., MEDLINE,CINAHL…):

PubMed

Interface

(i.e., Ovid, EBSCO…):

NA

Research Question

- To examine childhood cancer survival rates among children (0-19 years at diagnosis) with a migrant background compared to children with a native background.
- To explore the social determinants of health potentially associated with survival rates of childhood cancer.

PICO Format

(Outline the PICOs for your question — i.e., Patient, Intervention, Comparison, Outcome, and Study Design — as applicable)

**Population**	Children with migrant background (0-19yrs at time of diagnosis) who have been diagnosed with cancer
**I**	Not applicable
**C**	Children with non-migrant children (0-19yrs) who have been diagnosed with cancer
**O**	Childhood cancer survival rates
**S**	This review will focus on observational studies investigating childhood cancer survival rates. We will include longitudinal cohort, case-control, cross-sectional, and registry-based studies, along with mixed methods studies that combine quantitative and qualitative data.

Inclusion Criteria

(List criteria such as age groups, study designs, etc., to be included) *[optional]*

- Participants aged 0-19 years at diagnosis of childhood cancer.
- Studies including both children with migrant background and non-migrant children, where data for children can be separated.
- The studies include children who have a migrant background, defined as individuals who fall into the following categories:
  - Children who migrated to a new host country.
  - Children who were born in and are residing in a country that at least one of their parents previously entered as a migrant.
- Studies that have examined survival as a primary or secondary outcome measure for children with migrant background and non-migrant children separately (i.e., 1-, 5-, 10 years survival).
- Study Design:
  - Observational studies (longitudinal cohort, case-control, cross-sectional, registry-based); Mixed methods studies (combining quantitative and qualitative data); Completed studies with final results.
- Published from the year 2000 onwards.
- Studies from all global regions.

Exclusion Criteria

(List criteria such as study designs, date limits, etc., to be excluded) *[optional]*

- Studies including adults and children where data on children cannot be separated.
- Studies not reporting survival rates for both migrant and non-migrant children.
- Case reports, case series, editorials, opinions, non-peer-reviewed articles.
- Studies not directly examining childhood cancer survival rates.

Was a search filter applied?

No ■ Yes ☐

If YES, which one(s) (e.g., Cochrane RCT filter, PubMed Clinical Queries filter)? Provide the source if this is a published filter. *[mandatory if YES to previous question — textbox]*

Please copy and paste your search strategy here, exactly as run, including the number of hits per line. ***[mandatory]***

Search no	Search terms	Results
1	"Adolescent"[Mesh] OR "Adolescent Health"[Mesh] OR "Child"[Mesh] OR "Child Health"[Mesh] OR "Infant"[Mesh] OR "Infant Health"[Mesh] OR "Pediatrics"[Mesh]	4,079,365
2	adolescen*[Title/Abstract] OR babies[Title/Abstract] OR baby[Title/Abstract] OR boy[Title/Abstract] OR boyhood[Title/Abstract] OR boys[Title/Abstract] OR child[Title/Abstract] OR childhood*[Title/Abstract] OR children*[Title/Abstract] OR childs[Title/Abstract] OR girl*[Title/Abstract] OR infan*[Title/Abstract] OR juvenil*[Title/Abstract] OR kid[Title/Abstract] OR kids[Title/Abstract] OR paediatri*[Title/Abstract] OR pediatri*[Title/Abstract] OR preadolesc*[Title/Abstract] OR pre-adolesc*[Title/Abstract] OR preschool*[Title/Abstract] OR pre-school*[Title/Abstract] OR preteen*[Title/Abstract] OR pre-teen*[Title/Abstract] OR schoolchild*[Title/Abstract] OR teen*[Title/Abstract] OR toddler*[Title/Abstract] OR youth*[Title/Abstract]	2,753,513
3	#1 OR #2	4,817,833
4	"Cancer Survivors"[Mesh] OR "Neoplasms"[Mesh]	3,984,160
5	cancer*[Title/Abstract] OR carcinom*[Title/Abstract] OR craniopharyngiom*[Title/Abstract] OR ependymom*[Title/Abstract] OR gangliogliom*[Title/Abstract] OR glioblastom*[Title/Abstract] OR glioma*[Title/Abstract] OR hodgkins[Title/Abstract] OR "hodgkin s"[Title/Abstract] OR leukaemi*[Title/Abstract] OR leukemi*[Title/Abstract] OR lymphoma*[Title/Abstract] OR malignan*[Title/Abstract] OR medulloblastom*[Title/Abstract] OR neoplas*[Title/Abstract] OR nephroblastom*[Title/Abstract] OR neuroblastom*[Title/Abstract] OR oncolog*[Title/Abstract] OR osteosarcom*[Title/Abstract] OR retinoblastom*[Title/Abstract] OR rhabdomyosarcom*[Title/Abstract] OR sarcoma*[Title/Abstract] OR tumor*[Title/Abstract] OR tumour*[Title/Abstract] OR wilms[Title/Abstract]	4,636,178
6	#4 OR #5	5,425,123
7	"Survival Analysis"[Mesh] OR "Survival Rate"[Mesh]	493,507
8	"death rate*"[Title/Abstract] OR survival[Title/Abstract]	1,271,271
9	#7 OR #8	1,455,350
10	"Emigrants and Immigrants"[Mesh] OR "Ethnic and Racial Minorities"[Mesh] OR "Ethnicity"[Mesh] OR "Human Migration"[Mesh] OR "Refugees"[Mesh] OR "Transients and Migrants"[Mesh] OR "Neoplasms/ethnology"[Mesh]	179,988
11	asylee*[Title/Abstract] OR asylum*[Title/Abstract] OR "birth countr*"[Title/Abstract] OR "countr* of birth"[Title/Abstract] OR "countr* of origin"[Title/Abstract] OR diaspora[Title/Abstract] OR displaced[Title/Abstract] OR displacement*[Title/Abstract] OR emigra*[Title/Abstract] OR ethnic*[Title/Abstract] OR "foreign background*"[Title/Abstract] OR "foreign born"[Title/Abstract]	619,290

	OR "foreign citizen*"[Title/Abstract] OR "foreign national*"[Title/Abstract] OR "foreign origin*"[Title/Abstract] OR "foreign parent*"[Title/Abstract] OR foreignborn[Title/Abstract] OR foreigner*[Title/Abstract] OR "humanitarian entrant*"[Title/Abstract] OR immigra*[Title/Abstract] OR migrant*[Title/Abstract] OR minoritised[Title/Abstract] OR minoritized[Title/Abstract] OR "minority group*"[Title/Abstract] OR "minority patient*"[Title/Abstract] OR "minority status"[Title/Abstract] OR "naturali*ed citizen*"[Title/Abstract] OR "non citizen*"[Title/Abstract] OR "non native"[Title/Abstract] OR nonnative[Title/Abstract] OR race[Title/Abstract] OR racial*[Title/Abstract] OR refugee*[Title/Abstract] OR "second generation"[Title/Abstract] OR trafficked[Title/Abstract] OR transnational*[Title/Abstract] OR undocumented[Title/Abstract]	
12	migrat*[Title/Abstract] NOT ("cell migration"[Title/Abstract:~2] OR "invasion migration"[Title/Abstract:~2] OR "proliferation migration"[Title/Abstract:~2])	269,665
13	"Vulnerable Populations"[Mesh] OR "Financial Stress"[Mesh] OR "Neighborhood Characteristics"[Mesh] OR "Socioeconomic Disparities in Health"[Mesh] OR "Health Status Disparities"[Mesh] OR "Healthcare Disparities"[Mesh] OR "Health Inequities"[Mesh:NoExp] OR "Socioeconomic Factors"[Mesh]	559,989
14	disadvantag*[Title/Abstract] OR "economic* burden*"[Title/Abstract] OR "economic* challeng*"[Title/Abstract] OR "economic* hardship*"[Title/Abstract] OR "economic* strain*"[Title/Abstract] OR "economic* stress*"[Title/Abstract] OR "financial* burden*"[Title/Abstract] OR "financial* challeng*"[Title/Abstract] OR "financial hardship*"[Title/Abstract] OR "financial* strain*"[Title/Abstract] OR "financial* stress*"[Title/Abstract] OR gentrifi*[Title/Abstract] OR "health care disparit*"[Title/Abstract] OR "health disparit*"[Title/Abstract] OR "healthcare disparit*"[Title/Abstract] OR neighborhood*[Title/Abstract] OR neighbourhood*[Title/Abstract] OR "social determinant*"[Title/Abstract] OR "social disparit*"[Title/Abstract] OR "social equity"[Title/Abstract] OR "social factor*"[Title/Abstract] OR "social inequalit*"[Title/Abstract] OR "social risk*"[Title/Abstract] OR "socio demographic*"[Title/Abstract] OR "socio economic*"[Title/Abstract] OR sociodemographic*[Title/Abstract] OR socioeconomic*[Title/Abstract] OR underserved[Title/Abstract] OR "vulnerable group*"[Title/Abstract] OR "vulnerable population*"[Title/Abstract]	535,138
15	#10 OR #11 OR #12 OR #13 OR #14	1,756,549
16	#3 AND #6 AND #9 AND #15	4,628

PEER REVIEW ASSESSMENT: *THIS SECTION TO BE FILLED IN BY THE REVIEWER*

Reviewer: 1
Date completed: Aug 14th 2024

1. TRANSLATION

A ---No revisions	☐
B --- Revision(s) suggested	☐x
C --- Revision(s) required	☐

If "B" or "C," please provide an explanation or example:

Does the search strategy match the research question/PICO?

Strategy, concepts, PICO and RQ are well aligned. Except its not clear how you will assess social determinants of health. For example, you could assess social determinants of survival, by considering them as covariates, mediators or moderators in multivariable analyses. If you want to look at social determinants of survival, only in migrants, you can state this in your 'secondary' objective.

Are the search concepts clear?
Concepts are clear – children, cancer, survival, and migrant status.

Are there too many or too few PICO elements included?
PICO are clear and appropriate. You might consider specifying what you will do with the mixed methods studies – will you discount the qualitative findings and only extract survival outcomes?

Are the search concepts too narrow or too broad?
Concepts are appropriate.

Are unconventional or complex strategies explained?
Yes

Does the search retrieve too many or too few records? **(Please show number of hits per line.)**
Final number of results are high for one database. You could remove "social determinants" and "vulnerable populations" search terms, as some studies on social determinants may not assess migrant status.

You could edit migrant concept, considering asylum might return findings on psychiatric asylum by editing to: (asylum adj2 (seek* OR population*).

Expand free-text terms for survival: Mortality adj3 rate, proportional hazards, cox regression, recovery adj2 rate,

Are Boolean or proximity operators used correctly?
Yes, Term for "naturali*ed citizen" should use "naturali?ed citizen" to allow for various spellings.
"non citizen*" OR "non native" should use: "non?citizen*"[Title/Abstract] OR "non?native"

Could precision be improved by using proximity operators (eg, adjacent, near, within) or phrase searching instead of AND?
As above.

Is the width of proximity operators suitable (eg, might adj5 pick up more variants than adj2)? *Yes*

3. SUBJECT HEADINGS

A ---No revisions	☐X
B --- Revision(s) suggested	☐
C --- Revision(s) required	☐

If "B" or "C," please provide an explanation or example:

Are the subject headings relevant?

Are any subject headings too broad or too narrow?

Are subject headings exploded where necessary and vice versa?

Are major headings ("starring" or restrict to focus) used? If so, is there adequate justification?
-
Are subheadings missing?

Are subheadings attached to subject headings? (Floating subheadings may be preferred.)

Are floating subheadings relevant and used appropriately?

Are both subject headings and terms in free text (see the following) used for each concept?

4. TEXT WORD SEARCHING

A ---No revisions	☐X
B --- Revision(s)suggested	☐
C --- Revision(s) required	☐

If “B” or “C,” please provide an explanation or example:

Does the search include all spelling variants in free text (eg, UK vs. US spelling)? Yes.<br><br>*Does the search include all synonyms or antonyms (eg, opposites)? Yes*<br><br>*Does the search capture relevant truncation (ie, is truncation at the correct place)? Yes*<br><br>*Is the truncation too broad or too narrow? OK.*<br><br>*Are acronyms or abbreviations used appropriately? Do they capture irrelevant material? Are the full terms also included? Yes.*<br><br>*Have the appropriate fields been searched; for example, is the choice of the text word fields (.tw.) or all fields (.af.) appropriate? Are there any other fields to be included or excluded (database specific)? Yes.*<br><br>Should any long strings be broken into several shorter search statements?<br>-

5. SPELLING, SYNTAX, AND LINE NUMBERS

A ---No revisions	☐X
B --- Revision(s)suggested	☐
C --- Revision(s) required	☐

If “B” or “C,” please provide an explanation or example:

Are there any spelling errors? No<br><br>*Are there any errors in system syntax; for example, the use of a truncation symbol from a different search interface? No*<br><br>*Are there incorrect line combinations or orphan lines (ie, lines that are not referred to in the final summation that could indicate an error in an AND or OR statement)? No*

6. LIMITS AND FILTERS

A ---No revisions	☐X
B --- Revision(s) suggested	☐
C --- Revision(s) required	☐

If “B” or “C,” please provide an explanation or example:

OVERALL EVALUATION (Note: If one or more “revision required” is noted above, the response below must be “revisions required”.)

A ---No revisions	☐
B --- Revision(s) suggested	☐X
C --- Revision(s) required	☐

Additional comments:

Clear and appropriate strategy, with some suggested edits to align RQ, concepts and search terms. You may also want to search OVID: EMBASE.

PRESS Guideline — Search Submission & Peer Review Assessment

SEARCH SUBMISSION: *THIS SECTION TO BE FILLED IN BY THE SEARCHER*

Searcher: Vian Rajabzadeh **Email:** vian.rajabzadeh.uu.se
Reviewer: 2
Date submitted: Date requested by: 13th September 2024

Systematic Review Title:

Childhood Cancer Survival among Children with a Migrant Background: A Systematic Review Protocol

This search strategy is …

x	My PRIMARY (core) database strategy — First time submitting a strategy for search question and database
	My PRIMARY (core) strategy — Follow-up review NOT the first time submitting a strategy for search question and database. If this is a response to peer review, itemize the changes made to the review suggestions
	SECONDARY search strategy— First time submitting a strategy for search question and database
	SECONDARY search strategy — NOT the first time submitting a strategy for search question and database. If this is a response to peer review, itemize the changes made to the review suggestions

Database

(i.e., MEDLINE,CINAHL…):

PubMed

Interface

(i.e., Ovid, EBSCO…):

NA

Research Question

(Describe the purpose of the search)

- To examine childhood cancer survival rates among children (0-19 years at diagnosis) with a migrant background compared to children with a native background.
- To explore the social determinants of health potentially associated with survival rates of childhood cancer.

PICO Format

(Outline the PICOs for your question — i.e., Patient, Intervention, Comparison, Outcome, and Study Design — as applicable)

**Population**	Children with migrant background (0-19yrs at time of diagnosis) who have been diagnosed with cancer
**I**	Not applicable
**C**	Children with non-migrant children (0-19yrs) who have been diagnosed with cancer
**O**	Childhood cancer survival rates
**S**	This review will focus on observational studies investigating childhood cancer survival rates. We will include longitudinal cohort, case-control, cross-sectional, and registry-based studies, along with mixed methods studies that combine quantitative and qualitative data.

Inclusion Criteria

(List criteria such as age groups, study designs, etc., to be included) *[optional]*

- Participants aged 0-19 years at diagnosis of childhood cancer
- Studies including both children with migrant background and non-migrant children, where data for children can be separated.
- The studies include children who have a migrant background, defined as individuals who fall into the following categories:
  - Children who migrated to a new host country.
  - Children who were born in and are residing in a country that at least one of their parents previously entered as a migrant .
- Studies that have examined survival as a primary or secondary outcome measure for children with migrant background and non-migrant children separately (i.e., 1-, 5-, 10 years survival).
- Study Design:
  - Observational studies (longitudinal cohort, case-control, cross-sectional, registry-based); Mixed methods studies (combining quantitative and qualitative data); Completed studies with final results
- Published from the year 2000 onwards
- Studies from all global regions

Exclusion Criteria

(List criteria such as study designs, date limits, etc., to be excluded) *[optional]*

- Studies including adults and children where data on children cannot be separated.
- Studies not reporting survival rates for both migrant and non-migrant children.
- Case reports, case series, editorials, opinions, non-peer-reviewed articles
- Studies not directly examining childhood cancer survival rates

Was a search filter applied?

No ■ Yes ☐

If YES, which one(s) (e.g., Cochrane RCT filter, PubMed Clinical Queries filter)? Provide the source if this is a published filter. *[mandatory if YES to previous question — textbox]*

Please copy and paste your search strategy here, exactly as run, including the number of hits per line. ***[mandatory]***

Search no	Search terms	Results
1	"Adolescent"[Mesh] OR "Adolescent Health"[Mesh] OR "Child"[Mesh] OR "Child Health"[Mesh] OR "Infant"[Mesh] OR "Infant Health"[Mesh] OR "Pediatrics"[Mesh]	4,079,365
2	adolescen*[Title/Abstract] OR babies[Title/Abstract] OR baby[Title/Abstract] OR boy[Title/Abstract] OR boyhood[Title/Abstract] OR boys[Title/Abstract] OR child[Title/Abstract] OR childhood*[Title/Abstract] OR children*[Title/Abstract] OR childs[Title/Abstract] OR girl*[Title/Abstract] OR infan*[Title/Abstract] OR juvenil*[Title/Abstract] OR kid[Title/Abstract] OR kids[Title/Abstract] OR paediatri*[Title/Abstract] OR pediatri*[Title/Abstract] OR preadolesc*[Title/Abstract] OR pre-adolesc*[Title/Abstract] OR preschool*[Title/Abstract] OR pre-school*[Title/Abstract] OR preteen*[Title/Abstract] OR pre-teen*[Title/Abstract] OR schoolchild*[Title/Abstract] OR teen*[Title/Abstract] OR toddler*[Title/Abstract] OR youth*[Title/Abstract]	2,753,513
3	#1 OR #2	4,817,833
4	"Cancer Survivors"[Mesh] OR "Neoplasms"[Mesh]	3,984,160
5	cancer*[Title/Abstract] OR carcinom*[Title/Abstract] OR craniopharyngiom*[Title/Abstract] OR ependymom*[Title/Abstract] OR gangliogliom*[Title/Abstract] OR glioblastom*[Title/Abstract] OR glioma*[Title/Abstract] OR hodgkins[Title/Abstract] OR "hodgkin s"[Title/Abstract] OR leukaemi*[Title/Abstract] OR leukemi*[Title/Abstract] OR lymphoma*[Title/Abstract] OR malignan*[Title/Abstract] OR medulloblastom*[Title/Abstract] OR neoplas*[Title/Abstract] OR nephroblastom*[Title/Abstract] OR neuroblastom*[Title/Abstract] OR oncolog*[Title/Abstract] OR osteosarcom*[Title/Abstract] OR retinoblastom*[Title/Abstract] OR rhabdomyosarcom*[Title/Abstract] OR sarcoma*[Title/Abstract] OR tumor*[Title/Abstract] OR tumour*[Title/Abstract] OR wilms[Title/Abstract]	4,636,178
6	#4 OR #5	5,425,123
7	"Survival Analysis"[Mesh] OR "Survival Rate"[Mesh]	493,507
8	"death rate*"[Title/Abstract] OR survival[Title/Abstract]	1,271,271
9	#7 OR #8	1,455,350
10	"Emigrants and Immigrants"[Mesh] OR "Ethnic and Racial Minorities"[Mesh] OR "Ethnicity"[Mesh] OR "Human Migration"[Mesh] OR "Refugees"[Mesh] OR "Transients and Migrants"[Mesh] OR "Neoplasms/ethnology"[Mesh]	179,988
11	asylee*[Title/Abstract] OR asylum*[Title/Abstract] OR "birth countr*"[Title/Abstract] OR "countr* of birth"[Title/Abstract] OR "countr* of origin"[Title/Abstract] OR diaspora[Title/Abstract] OR displaced[Title/Abstract] OR displacement*[Title/Abstract] OR emigra*[Title/Abstract] OR ethnic*[Title/Abstract] OR "foreign background*"[Title/Abstract] OR "foreign born"[Title/Abstract]	619,290

	OR "foreign citizen*"[Title/Abstract] OR "foreign national*"[Title/Abstract] OR "foreign origin*"[Title/Abstract] OR "foreign parent*"[Title/Abstract] OR foreignborn[Title/Abstract] OR foreigner*[Title/Abstract] OR "humanitarian entrant*"[Title/Abstract] OR immigra*[Title/Abstract] OR migrant*[Title/Abstract] OR minoritised[Title/Abstract] OR minoritized[Title/Abstract] OR "minority group*"[Title/Abstract] OR "minority patient*"[Title/Abstract] OR "minority status"[Title/Abstract] OR "naturali*ed citizen*"[Title/Abstract] OR "non citizen*"[Title/Abstract] OR "non native"[Title/Abstract] OR nonnative[Title/Abstract] OR race[Title/Abstract] OR racial*[Title/Abstract] OR refugee*[Title/Abstract] OR "second generation"[Title/Abstract] OR trafficked[Title/Abstract] OR transnational*[Title/Abstract] OR undocumented[Title/Abstract]	
12	migrat*[Title/Abstract] NOT ("cell migration"[Title/Abstract:~2] OR "invasion migration"[Title/Abstract:~2] OR "proliferation migration"[Title/Abstract:~2])	269,665
13	"Vulnerable Populations"[Mesh] OR "Financial Stress"[Mesh] OR "Neighborhood Characteristics"[Mesh] OR "Socioeconomic Disparities in Health"[Mesh] OR "Health Status Disparities"[Mesh] OR "Healthcare Disparities"[Mesh] OR "Health Inequities"[Mesh:NoExp] OR "Socioeconomic Factors"[Mesh]	559,989
14	disadvantag*[Title/Abstract] OR "economic* burden*"[Title/Abstract] OR "economic* challeng*"[Title/Abstract] OR "economic* hardship*"[Title/Abstract] OR "economic* strain*"[Title/Abstract] OR "economic* stress*"[Title/Abstract] OR "financial* burden*"[Title/Abstract] OR "financial* challeng*"[Title/Abstract] OR "financial hardship*"[Title/Abstract] OR "financial* strain*"[Title/Abstract] OR "financial* stress*"[Title/Abstract] OR gentrifi*[Title/Abstract] OR "health care disparit*"[Title/Abstract] OR "health disparit*"[Title/Abstract] OR "healthcare disparit*"[Title/Abstract] OR neighborhood*[Title/Abstract] OR neighbourhood*[Title/Abstract] OR "social determinant*"[Title/Abstract] OR "social disparit*"[Title/Abstract] OR "social equity"[Title/Abstract] OR "social factor*"[Title/Abstract] OR "social inequalit*"[Title/Abstract] OR "social risk*"[Title/Abstract] OR "socio demographic*"[Title/Abstract] OR "socio economic*"[Title/Abstract] OR sociodemographic*[Title/Abstract] OR socioeconomic*[Title/Abstract] OR underserved[Title/Abstract] OR "vulnerable group*"[Title/Abstract] OR "vulnerable population*"[Title/Abstract]	535,138
15	#10 OR #11 OR #12 OR #13 OR #14	1,756,549
16	#3 AND #6 AND #9 AND #15	4,628

PEER REVIEW ASSESSMENT: *THIS SECTION TO BE FILLED IN BY THE REVIEWER*

Reviewer: 2
Date completed: 13/9/2024

1. TRANSLATION

A ---No revisions	x☐
B --- Revision(s) suggested	☐
C --- Revision(s) required	☐

If "B" or "C," please provide an explanation or example:

Does the search strategy match the research question/PICO?

Are the search concepts clear?

Are there too many or too few PICO elements included?

Are the search concepts too narrow or too broad?

Does the search retrieve too many or too few records? (Please show number of hits per line.)

Are unconventional or complex strategies explained?

2. BOOLEAN AND PROXIMITY OPERATORS

A ---No revisions	☐
B --- Revision(s) suggested	x☐
C --- Revision(s) required	☐

If "B" or "C," please provide an explanation or example:

Are Boolean or proximity operators used correctly?
Yes

Could precision be improved by using proximity operators (eg, adjacent, near, within) or phrase searching instead of AND?
I suggest you also search for "haematological malignancies" (includes leukemias & lymphomas) as well as "brain tumours" and "solid tumours" which are the 3 common groups of diagnosis. "Pediatric malignancies" could also be a term used for all kinds of such diagnosis.

Is the width of proximity operators suitable (eg, might adj5 pick up more variants than adj2)?
Yes

3. SUBJECT HEADINGS

A ---No revisions	x
B --- Revision(s) suggested	
C --- Revision(s) required	

If “B” or “C,” please provide an explanation or example:

Are the subject headings relevant?

Are any subject headings too broad or too narrow?

Are subject headings exploded where necessary and vice versa?

Are major headings (“starring” or restrict to focus) used? If so, is there adequate justification?
-
Are subheadings missing?

Are subheadings attached to subject headings? (Floating subheadings may be preferred.)

Are floating subheadings relevant and used appropriately?

Are both subject headings and terms in free text (see the following) used for each concept?

4. TEXT WORD SEARCHING

A ---No revisions	x
B --- Revision(s)suggested	
C --- Revision(s) required	

If “B” or “C,” please provide an explanation or example:

Does the search include all spelling variants in free text (eg, UK vs. US spelling)?<br><br>*Does the search include all synonyms or antonyms (eg, opposites)?*<br><br>*Does the search capture relevant truncation (ie, is truncation at the correct place)?*<br><br>*Is the truncation too broad or too narrow?*<br><br>*Are acronyms or abbreviations used appropriately? Do they capture irrelevant material? Are the full terms also included?*<br><br>*Have the appropriate fields been searched; for example, is the choice of the text word fields (.tw.) or all fields (.af.) appropriate? Are there any other fields to be included or excluded (database specific)?*<br><br>Should any long strings be broken into several shorter search statements?<br>-

5. SPELLING, SYNTAX, AND LINE NUMBERS

A ---No revisions	x
B --- Revision(s)suggested	
C --- Revision(s) required	

If “B” or “C,” please provide an explanation or example:

Are there any spelling errors?<br><br>*Are there any errors in system syntax; for example, the use of a truncation symbol from a different search interface?*<br><br>*Are there incorrect line combinations or orphan lines (ie, lines that are not referred to in the final summation that could indicate an error in an AND or OR statement)?*

6. LIMITS AND FILTERS

A ---No revisions	x
B --- Revision(s) suggested	
C --- Revision(s) required	

If “B” or “C,” please provide an explanation or example:

OVERALL EVALUATION (Note: If one or more “revision required” is noted above, the response below must be “revisions required”.)

A ---No revisions	
B --- Revision(s) suggested	x
C --- Revision(s) required	

Additional comments:

Supplementary Appendix 3: PubMed search strategy

PubMed, 2024-09-17			
Search no	Search terms	Results	Comments
1	"Adolescent"[Mesh] OR "Adolescent Health"[Mesh] OR "Child"[Mesh] OR "Child Health"[Mesh] OR "Infant"[Mesh] OR "Infant Health"[Mesh] OR "Pediatrics"[Mesh]	4,107,878	
2	adolescen*[Title/Abstract] OR babies[Title/Abstract] OR baby[Title/Abstract] OR boy[Title/Abstract] OR boyhood[Title/Abstract] OR boys[Title/Abstract] OR child[Title/Abstract] OR childhood*[Title/Abstract] OR children*[Title/Abstract] OR childs[Title/Abstract] OR girl*[Title/Abstract] OR infan*[Title/Abstract] OR juvenil*[Title/Abstract] OR kid[Title/Abstract] OR kids[Title/Abstract] OR paediatri*[Title/Abstract] OR pediatri*[Title/Abstract] OR preadolesc*[Title/Abstract] OR pre-adolesc*[Title/Abstract] OR preschool*[Title/Abstract] OR pre-school*[Title/Abstract] OR preteen*[Title/Abstract] OR pre-teen*[Title/Abstract] OR schoolchild*[Title/Abstract] OR teen*[Title/Abstract] OR toddler*[Title/Abstract] OR youth*[Title/Abstract]	2,784,776	
3	#1 OR #2	4,860,146	
4	"Cancer Survivors"[Mesh] OR "Neoplasms"[Mesh]	4,018,245	
5	cancer*[Title/Abstract] OR carcinom*[Title/Abstract] OR craniopharyngiom*[Title/Abstract] OR ependymom*[Title/Abstract] OR gangliogliom*[Title/Abstract] OR glioblastom*[Title/Abstract] OR glioma*[Title/Abstract] OR hodgkins[Title/Abstract] OR "hodgkin s"[Title/Abstract] OR leukaemi*[Title/Abstract] OR leukemi*[Title/Abstract] OR lymphoma*[Title/Abstract] OR malignan*[Title/Abstract] OR medulloblastom*[Title/Abstract] OR neoplas*[Title/Abstract] OR nephroblastom*[Title/Abstract] OR neuroblastom*[Title/Abstract] OR oncolog*[Title/Abstract] OR	4,694,746	

	osteosarcom*[Title/Abstract] OR retinoblastom*[Title/Abstract] OR rhabdomyosarcom*[Title/Abstract] OR sarcoma*[Title/Abstract] OR tumor*[Title/Abstract] OR tumour*[Title/Abstract] OR wilms[Title/Abstract]		
6	#4 OR #5	5,486,756	
7	"Survival Analysis"[Mesh] OR "Survival Rate"[Mesh]	496,790	
8	"cox regression*"[Title/Abstract] OR "death rate*"[Title/Abstract] OR "mortality rate"[Title/Abstract:~3] OR "mortality rates"[Title/Abstract:~3] OR "proportional hazard*"[Title/Abstract] OR "recovery rate"[Title/Abstract:~2] OR "recovery rates"[Title/Abstract:~2] OR survival[Title/Abstract]	1,584,307	
9	#7 OR #8	1,728,008	
10	"Emigrants and Immigrants"[Mesh] OR "Ethnic and Racial Minorities"[Mesh] OR "Ethnicity"[Mesh] OR "Human Migration"[Mesh] OR "Refugees"[Mesh] OR "Transients and Migrants"[Mesh] OR "Neoplasms/ethnology"[Mesh]	181,923	
11	asylee*[Title/Abstract] OR asylum*[Title/Abstract] OR "birth countr*"[Title/Abstract] OR "countr* of birth"[Title/Abstract] OR "countr* of origin"[Title/Abstract] OR diaspora[Title/Abstract] OR displaced[Title/Abstract] OR displacement*[Title/Abstract] OR emigra*[Title/Abstract] OR ethnic*[Title/Abstract] OR "foreign background*"[Title/Abstract] OR "foreign born"[Title/Abstract] OR "foreign citizen*"[Title/Abstract] OR "foreign national*"[Title/Abstract] OR "foreign origin*"[Title/Abstract] OR "foreign parent*"[Title/Abstract] OR foreignborn[Title/Abstract] OR foreigner*[Title/Abstract] OR "humanitarian entrant*"[Title/Abstract] OR immigra*[Title/Abstract] OR migrant*[Title/Abstract] OR minoritised[Title/Abstract] OR minoritized[Title/Abstract] OR "minority group*"[Title/Abstract] OR "minority patient*"[Title/Abstract] OR "minority status"[Title/Abstract] OR "naturali*ed citizen*"[Title/Abstract] OR "non citizen*"[Title/Abstract] OR "non native"[Title/Abstract] OR nonnative[Title/Abstract] OR race[Title/Abstract] OR racial*[Title/Abstract] OR refugee*[Title/Abstract] OR "second generation"[Title/Abstract] OR trafficked[Title/Abstract] OR transnational*[Title/Abstract] OR undocumented[Title/Abstract]	628,717	
12	migrat*[Title/Abstract] NOT ("cell migration"[Title/Abstract:~2] OR "invasion migration"[Title/Abstract:~2] OR "proliferation migration"[Title/Abstract:~2])	273,085	
13	"Vulnerable Populations"[Mesh] OR "Financial Stress"[Mesh] OR "Neighborhood Characteristics"[Mesh] OR "Socioeconomic Disparities in Health"[Mesh] OR "Health Status Disparities"[Mesh] OR "Healthcare Disparities"[Mesh] OR "Health Inequities"[Mesh:NoExp] OR "Socioeconomic Factors"[Mesh]	564,239	
14	disadvantag*[Title/Abstract] OR "economic* burden*"[Title/Abstract] OR "economic* challeng*"[Title/Abstract] OR "economic* hardship*"[Title/Abstract] OR "economic*	546,941	

	strain*"[Title/Abstract] OR "economic* stress*"[Title/Abstract] OR "financial* burden*"[Title/Abstract] OR "financial* challeng*"[Title/Abstract] OR "financial hardship*"[Title/Abstract] OR "financial* strain*"[Title/Abstract] OR "financial* stress*"[Title/Abstract] OR gentrifi*[Title/Abstract] OR "health care disparit*"[Title/Abstract] OR "health disparit*"[Title/Abstract] OR "healthcare disparit*"[Title/Abstract] OR neighborhood*[Title/Abstract] OR neighbourhood*[Title/Abstract] OR "social determinant*"[Title/Abstract] OR "social disparit*"[Title/Abstract] OR "social equity"[Title/Abstract] OR "social factor*"[Title/Abstract] OR "social inequalit*"[Title/Abstract] OR "social risk*"[Title/Abstract] OR "socio demographic*"[Title/Abstract] OR "socio economic*"[Title/Abstract] OR sociodemographic*[Title/Abstract] OR socioeconomic*[Title/Abstract] OR underserved[Title/Abstract] OR "vulnerable group*"[Title/Abstract] OR "vulnerable population*"[Title/Abstract]		
15	#10 OR #11 OR #12 OR #13 OR #14	1,781,159	
16	#3 AND #6 AND #9 AND #15	5,638	

Supplementary Appendix 4: Extraction sheet form

**Study Identification Features**

Study ID/record number	
Study title	
Authors' names	
Year of publication	
Citation	
DOI	
URL link	
Publication type or Source of data (e.g., journal or report)	
Abstract	
**Study Characteristics**	
Aim of the study	
Setting (e.g., geographical region)	
Study design (e.g., cohort study, cross-sectional, registry-based studies)	
Study duration or follow-up	
Population age	
Sample size	
Sampling method	
Method of data analysis	
Recruitment method	
Recruitment setting	
Type on consent	
Reported survival rates (1-, 2-, 3-, 5-, 10-year survival rates)	
**Population characteristics**	
Sex (male to female ratio or percentage)	
Age at diagnosis (mean age, age range)	
Country of population	
Type of cancer	
The distribution of cancer	
Race, ethnicity	
Type of migration	
Country of origin	
**Outcome summary**	
Survival rate year (1,2,3,5, and 10-year survival rates)	
Hazard ratio (migrant versus non-migrant background)	
Hazard ratio confidence interval	
Mortality rates	
Data extracted from Kaplan-Meier curves and other relevant metrics	
**Social determinants of health summary (as defined by the Centers for Disease Control and Prevention (CDC) framework**	

Economic Stability (e.g., income, poverty)	
Education (e.g., access to quality education)	
Social Support Networks (e.g., family, friends)	
Neighbourhood Environment (e.g., safety, quality housing)	
Healthcare Access (e.g., affordability, quality)	
